# *p*-Cymene Complexes of Ruthenium(II) as Antitumor Agents

**DOI:** 10.3390/molecules25215063

**Published:** 2020-10-31

**Authors:** María Angeles Pujante-Galián, Sergio A. Pérez, Mercedes G. Montalbán, Guzmán Carissimi, Marta G. Fuster, Gloria Víllora, Gabriel García

**Affiliations:** 1 Inorganic Chemistry Department, Faculty of Chemistry, Regional Campus of International Excellence “Campus Mare Nostrum”, University of Murcia, 30071 Murcia, Spain; mariaangeles.pujante@um.es (M.A.P.-G.); ggarcia@um.es (G.G.); 2 Chemical Engineering Department, Faculty of Chemistry, Regional Campus of International Excellence “Campus Mare Nostrum”, University of Murcia, 30071 Murcia, Spain; sa.perezhenarejos@um.es (S.A.P.); guzmanaugusto.carissimin@um.es (G.C.); marta.g.f@um.es (M.G.F.); gvillora@um.es (G.V.)

**Keywords:** ruthenium, complexes, cytotoxicity, anticancer activity, MTT assay

## Abstract

In this work, the cytotoxic behavior of six ruthenium(II) complexes of stoichiometry [(η^6^-*p*-cymene)RuCl_2_L] (I-VI), L = 4-cyanopyridine (I), 2-aminophenol (II), 4-aminophenol (III), pyridazine (IV), and [(*η*^6^-*p*-cymene)RuClL_2_]PF_6_; L = cyanopyridine (V), L = 2-aminophenol(VI) towards three cell lines was studied. Two of them, HeLa and MCF-7, are human carcinogenic cells from cervical carcinoma and human breast cancer, respectively. A comparison with healthy cells was carried out with BGM cells which are monkey epithelial cells of renal origin. The behavior of complex II exhibits selectivity towards healthy cells, which is a promising feature for use in cancer treatment since it might reduce the side effects of most current therapies.

## 1. Introduction

**Scheme 1:**
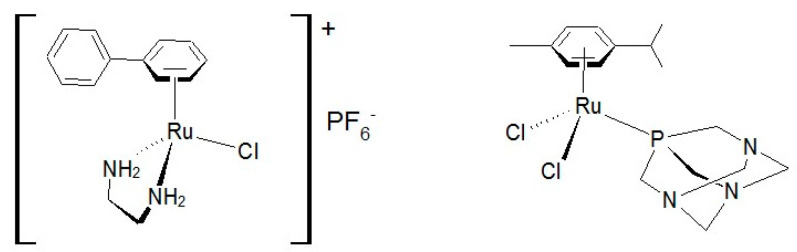
Complexes [*η*^6^-(biphenyl)Ru(en)Cl]PF_6_ [17,18,19,20,21] and [(*η*^6^-*p*-cymene)Ru(pta)Cl_2_] [22,23].

**Scheme 2:**
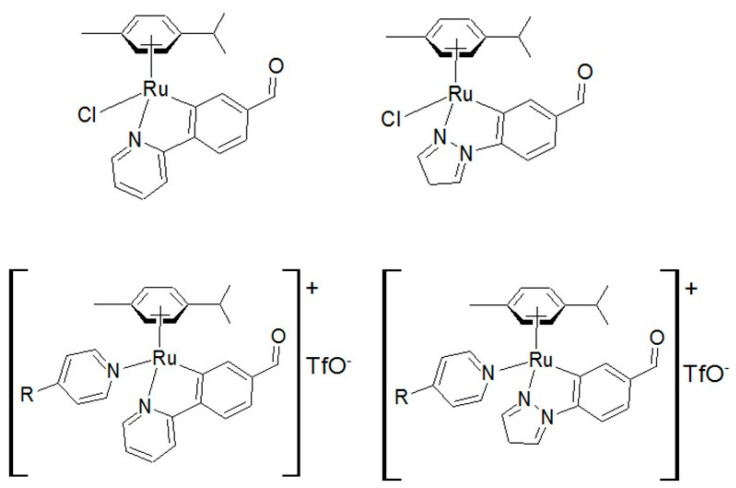
Complexes [(*η*^6^-*p*-cymene)Ru(CN)X]^0/+^ (X=Cl, py or 4-NMe_2_py) containing a cyclometalated 2-ppy or 1-ppz with a non-coordinated CHO [24].

Platinum complexes such as cis-platinum, carboplatin and oxaliplatin have long been used as anticancer drugs [1]. Recently [2], both bifunctional and monofunctional platinum drugs have been presented, not only in platinum dichloride(II) complexes, but also in more innovative platinum structures, such as cationic ones, which have been shown to be cytotoxic against aggressive tumors and orphaned in treatment. Ruthenium complexes are considered as potential replacements for platinum compounds in oncotherapy, although it is known [3] that ruthenium complexes have biological targets other than DNA, such as some enzymatic systems involved in tumor progression. In the design of new anticancer drugs [4,5,6,7,8,9], ruthenium complexes have raised great interest and have been tested against a number of cancer cell lines [10,11,12,13,14,15,16] and are regarded as promising candidates due to their unique and versatile biochemical properties. Thus, the half-sandwich complexes [*η*^6^-(biphenyl)Ru(en)Cl]PF_6_ [17,18,19,20,21] and [(*η*^6^-*p*-cymene)Ru(pta)Cl_2_] [22,23] represent an interesting new class of arene compounds with antitumor activity (Scheme 1). Recently, Ruiz et al. [24] described the synthesis of ruthenium (II) complexes containing *p*-cymene and ortho metalized ligands [(*η*^6^-*p*-cymene)Ru(CN)X]^0/+^ (X=Cl, py or 4-NMe_2_py) containing a cyclometalated 2-ppy or 1-ppz with a non-coordinated CHO group, and found that these compounds also present antitumor reactivity (Scheme 2).

In 1995, we described the synthesis and characterization of ruthenium(II) complexes containing the fragment *η*^6^-arene-Ru(II) (*η*^6^-arene = benzene or *p*-cymene), and potentially bidentate ligands of stoichiometry [(*η*^6^-*p*-cymene)RuCl_2_L] (L = 4-cyanopyridine (I) and 2-aminophenol (II) [25]. In this work, the synthesis and characterization of the ionic compound [Ru(*p*-cymene)Cl(2-aminophenol)2]Cl was also described. In a similar work, we obtained rhodium (III) compounds containing, in this case, the fragment Cp*Rh (Cp*=C_5_Me_5_) [26]. Subsequently, it has been shown that the ruthenium(II) compound we described, [(*η*^6^-*p*-cymene)Ru(o-phenylenediamine)Cl]PF_6_ [25], behaves as an antitumor agent against different kind of tumors [27,28]. Complex III was synthesized according to reference [29]. Furthermore, Pandey et al., in 1998 [30], described the structure obtained by X-ray diffraction of the [Ru(*p*-cymene) Cl (4-cyanopyridine)] complex, as well as the synthesis and characterization of the species with 4-cyanopyridine bridge: Ru(*p*-cymene)Cl2] (μ-4-cyanopyridine) [30] and in 2007, they also described the behavior of the mononuclear complex with terminal pyridine against DNA [31].

Furthermore, ruthenium compounds have low levels of toxicity and can be tolerated in vivo. Their advantages over platinum-based complexes include their various oxidation states, reaction mechanism, and different ligand substitution kinetics, thereby making them suitable for use in biological applications. Several studies have focused attention on the interaction between ruthenium complexes and their biological targets [32]. For example, Weiss et al. [33] developed a series of organometallic ruthenium(II)–arene complexes that exerted antimetastatic activity and lowered primary tumor growth. They demonstrated that the prototype compound, [Ru(*ɳ*^6^-*p*-cymene)Cl_2_(pta)], where pta = 1,3,5-triaza-7-phosphaadamantane (RAPTA-C), reduces the expansion of primary tumors in preclinical models for colorectal and ovarian carcinomas The organoruthenium compounds formed from Ru(II)(*η*^6^-*p*-cymene) chloride moieties and oxicam-based ligands have also been studied [34]. The aim of the mentioned work was to combine the anti-inflammatory properties of oxicams, a versatile family of heterocyclic compounds, and the anticancer activity of Ru(II)(arene) complexes. By means of in vitro assays, it was established that the complexes were active against the colon carcinoma HCT116 and breast cancer MDA MB 231 cancer cell lines. The cytotoxicity was found to be strongly dependent on the lipophilicity of the compound, as the most lipophilic compound was the most active in HCT116 cells. Moreover, the ruthenium(II) *p*-cymene complexes of naphthoquinone derivatives [Ru(II)(*ɳ^6^-p*-cymene)(Lap)(PTA)](PF_6_), and [Ru(II)(*ɳ*^6^-*p*-cymene)(Jug)(PTA)](PF_6_), 4 (Lap: lapachol, Plum: plumbagin, Law: lawsone, Jug: juglone, PTA: 1,3,5-triaza-7-phosphaadamantane) showed in vitro antiproliferative activity against human melanoma A375, liver hepatocellular carcinoma HepG-2, breast MCF-7, colon adenocarcinoma LoVo, ovary A2780 and colon carcinoma HCT-8 cancer cell lines under hypoxic conditions [35].

Recently, other half-sandwich ruthenium compounds with the general formula [Ru(*p*-cymene)(L-*N,N*)Cl][CF_3_SO_3_] (L = 3,6-di-2-pyridyl-1,2,4,5-tetrazine (**1**) and 6,7-dimethyl-2,3-bis(pyridin-2-yl)quinoxaline (**2**), obtained from the precursor dimer [Ru(*p*-cymene)(Cl)(μ-Cl)], have been assessed against human tumor cells: ovarian carcinoma A2780 and breast MCF7 and MDAMB231 adenocarcinoma cells and against normal primary fibroblasts. Compound 1 showed moderate cytotoxic activity, while compound 2 showed lower activity than previously reported for Ru(*p*-cymene) complexes [36]. In addition, the cytotoxic effects of new pyrazole carbothioamide derivatives and their four arene–ruthenium complexes were evaluated, using the MTT assay, against three cancer cell lines (HL-60, NALM-6 and WM-115) and normal human foreskin fibroblasts (HFF-1). It was found that the new arene–ruthenium(II) compounds inhibit the proliferation of cancer cells and protect patients against malignant wound infections due to their antimicrobial properties [8].

Ru(II) complexes are known to enter cells through multiple mechanisms, such as passive diffusion, active transport, and endocytosis [37]. However, it was noted that most nanostructured ruthenium complexes enter cells by endocytosis [38], although, the changes in ligands and hydrophobicity can modulate uptake and cellular localization. On the other hand, most Ru(II) complexes are known to have high selectivity for binding to DNA [39,40,41,42] and can also bind to DNA via interaction with aromatic ligands.

In this context, we studied the synthesis of complexes similar to those previously described by us, containing the Ru(*η*^6^-*p*-cymene) fragment, starting from the dimer [Ru(*η*^6^-*p*-cymene)(μ-Cl)Cl]2 [43] and different ligands in order to investigate the possible antitumor activity of these complexes. The compounds were characterized by C, H and N elemental analysis, infrared spectroscopy, proton nuclear magnetic resonance and high-resolution mass spectrometry. The cell lines chosen to evaluate the cytotoxic activity of the synthesized compounds were HeLa, MCF-7, and BGM cells, human carcinogenic cells from cervical carcinoma and human breast cancer and monkey epithelial cells of renal origin, respectively. The cytotoxicity was determined using the 3-(4,5-dimethylthiazol-2-yl)-2,5-diphenyltetrazolium bromide (MTT) assay.

## 2. Results and Discussion

**Scheme 3:**
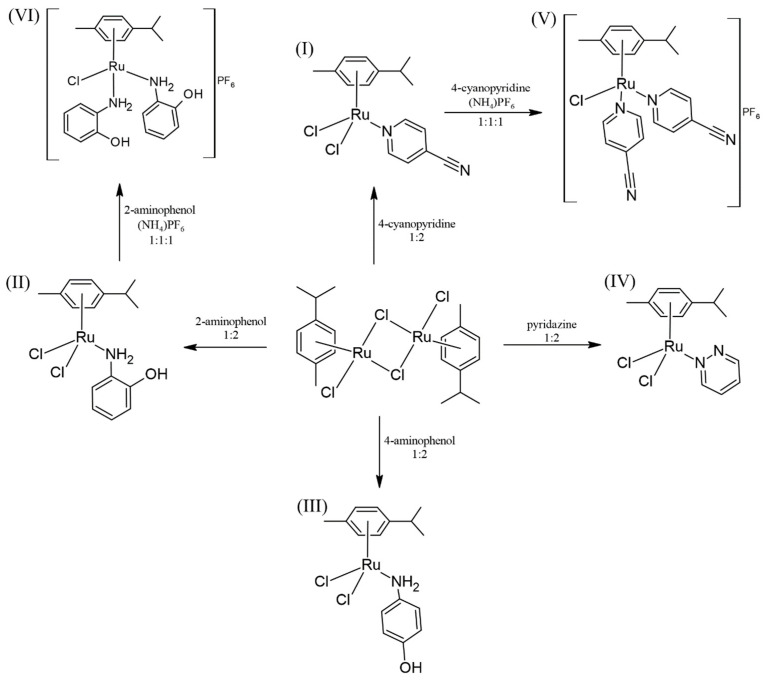
Scheme of reactions.

The compounds used as anticancer agents are summarized in the following scheme (Scheme 3).

From the *p*-cymene dimer, by the addition of the corresponding ligands in dichloromethane, the complexes I, II, III and IV were achieved. Compounds V and VI were obtained from I and II, the corresponding ligand, NH_4_PF_6,_ and a small amount of water. It is worth mentioning that the yields of the neutral compounds III and IV are higher than those of the ionic ones, V, but mainly VI, probably because the presence of water makes the reaction medium more ionic, dissolving and dragging part of the NH_4_PF_6__._

All the isolated ruthenium compounds are air-stable solids and gave satisfactory partial elemental analyses and their colors, yields, mass spectrometry and decomposition points are listed in Table 1. All these data are consistent with the proposed formula.

Infrared spectroscopy confirmed that the ligand acted as a monodentate even when the reaction conditions or proportions where changed. Thus, in all cases, ruthenium(II) prefers to be coordinated by pyridine or amine nitrogen acting as a monodentate ligand. This behavior has been observed previously [25].

The ruthenium(II) compounds used as antitumor agents are of two types: neutral and cationic. In the case of the cationic, the stabilizing anion was PF_6_^−^, which is easily distinguished both by infrared spectroscopy (due to the presence of two bands at, approximately, 840 and 560 cm^−1^, see Table 2) and by mass spectrometry (due to the presence of the peak corresponding to the anion at 145). In all the cases, the signals corresponding to ν (Ru–Cl) appear in the 265–300 range.

The new compounds were also characterized by NMR-^1^H and Cosy ^1^H-^1^H. The observed signals in Table 3 are consistent with the predicted values. The 1H NMR spectra of complexes III, IV and V are attached in Figures S1 to S3 of the Supporting Information as well as the 1H–1H COSY of IV (Figure S4). The solvent used in each case can be observed. The mass spectra of III, IV, and V are also attached in Figures S5 to S7.

The assignment of the aromatic protons of complex IV was done by COSY ^1^H-^1^H. In any case, the presence of isomers in the anionic species was observed.

### Cytotoxicity of the Complexes and the Ligand

**Figure 1:**
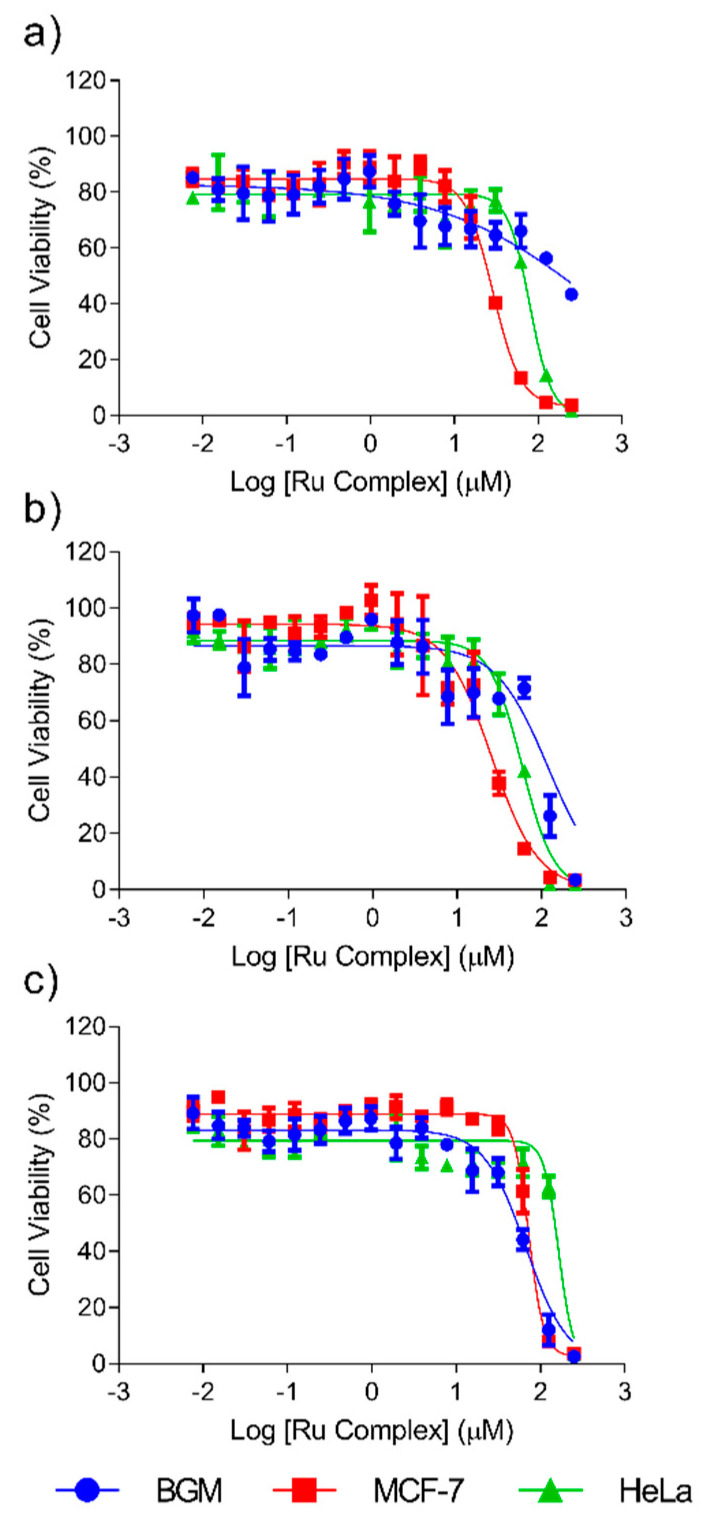
Dose–response curves of HeLa, MCF-7 and BGM cells treated with complexes II (**a**), VI (**b**) and III (**c**) for 48 h.

In vitro cytotoxicity tests of the synthesized ruthenium(II) compounds were carried out to establish their potential anticancer activities and to select the most active in this respect but, at the same time, the least harmful for healthy cells. A colorimetric MTT assay, that assesses cell metabolic activity, was used to determine cytotoxicity. This test is cost-effective, convenient and rapid [44]. The cytotoxicity of the complexes was seen to be strongly influenced by the chosen cell lines and by the structural features such as the ligand used. The IC_50_ values of HeLa, MCF-7 and BGM cells after exposure to a series of ruthenium(II) compounds for 48 h were calculated using a dose–response model, which was obtained from sigmoidal fitting of dose–response curves, as stated in the Experimental Section. The calculated IC_50_ values of the compounds and free ligands are shown in Table 4 and Table 5, respectively. The IC_50_ values of the ruthenium(II) compounds synthesized were compared in Table 4 with the values of cisplatin obtained from the literature [45,46] for the same cell lines. All complexes (except complex VI with Hela) were found to be less toxic than cisplatin with the cancer cell lines. Nevertheless, cisplatin is much more aggressive towards the BGM healthy cells. The differential selectivity of an anticancer drug towards cancer cells versus normal cells increases the likelihood of tumor-specific cytotoxicity, reducing the side effects in patients. The corresponding dose–response curves of Complexes II, III and VI are represented in Figure 1.

The results showed that ligands 4-cyanopyridine, pyridazine and 4-aminophenol do not contribute to the cytotoxic behavior of ruthenium (II) compounds (IC_50_ > 250 μM) on any of the three cell lines studied. The ligand 2-aminophenol only presents a cytotoxic effect against MCF-7 cells, but much lower than the ruthenium (II) complexes. The cytotoxic potency of Complexes I, IV and V is also negligible (IC_50_ > 250 μM) on the three cell lines studied. However, Complexes II, III and VI (see Figure 1) were cytotoxic in the case of at least two cell lines. Complexes II and VI (both with the ligand L = 2-aminophenol) were the most cytotoxic towards cancer cells both being more aggressive to MCF-7 breast cancer cells than towards cervical cancer cells HeLa. Of these two complexes, Complex II must be considered the best choice for cancer therapy because it was not cytotoxic towards the healthy cells used in this study (BGM), when IC_50_ values higher than 250 μM were reached. By contrast, Complex VI, while it had the stronger cytotoxic effect against tumor cells, showed lower selectivity between the tumor and healthy cells, with a cytotoxicity of 95 μM in BGM cells. Finally, Complex III (with the ligand 4-aminophenol) was more cytotoxic towards healthy cells than cancer cells, which rules out its suitability for the purpose of this work. Therefore, it seems that position 2 in the ligand is preferred for cytotoxic behavior. The above described behavior of Complex II is highly promising because its selectivity in the face of tumor cells is higher than most of the tumor used in current cancer therapies.

## 3. Materials and Methods

The solvents were dried by conventional methods. The ligands were commercial grade chemicals and [{(*p*-cymene)RuCl_2_}_2_] were prepared by published methods [23]. ^1^H NMR spectra were recorded on a Bruker Avance 200, 300 and 400 MHz instrument. IR spectra were recovered on a 100 FTIR Spectrometer as nujol mulls. The C, H and N analyses were obtained with a LECO CNHS-932 elemental microanalyzer. Thermal decomposition studies were carried out on a TGA–DTA TA Instruments. High resolution (HR)–ESI–MS spectrometry was obtained using a MS TOF Agilent Model 6220 spectrometer.

### 3.1. Synthesis of the Complexes

#### 3.1.1. Complexes I, II, III and IV

These were prepared according to the following procedure. To a dichloromethane (15 mL) solution of [{(*p*-cymene)RuCl_2_}_2_] (0.4902 mmol), the appropriate ligand (0.9804 mmol for I, II, III and IV) was added. The resulting suspension was stirred for 1 h and was concentrated. The solid obtained was separated by filtration, and repeatedly washed with diethyl ether. Complex III, as mentioned, was synthesized according to reference [29].

#### 3.1.2. Complexes V and VI

These were prepared according to the following procedure. To an ethanol (7 mL) solution of the respective compound II and IV, respectively, (0.6098 mmol) the corresponding ligand (0.6098 mmol), and NH_4_PF_6_ (0.6098 mmol) respective ligand (0.6098 mmol) were added. The mixture was stirred for 5 min and then, water (1.5 mL), was added. The solution darkened immediately and was stirred for 1 h. Partial evaporation of the solvent and the subsequent addition of diethyl ether caused the formation of a precipitate, which was filtered off and air-dried. These complexes were recrystallized from ethanol–diethyl ether.

### 3.2. Cell lines and Culture Media

Human cervical cancer cells (HeLa), human breast cancer cells (MCF-7) and green monkey kidney epithelial cells (BGM) were acquired from the American Type Culture Collection (ATCC, USA). The reason to choose human cervical cancer cells (HeLa) and human breast cancer cells (MCF-7) was to use a cell culture model that closely represented the human in vivo situation. The green monkey kidney epithelial cells (BGM) were chosen to compare cancer cells with healthy cells. Cell lines were maintained in Dulbecco’s Modified Eagle Medium (DMEM) with a low glucose content (1 g/L) supplemented with 10% (*v/v*) fetal bovine serum (FBS), 1 mM glutamax, 1% antibiotics (penicillin–streptomycin) and 1 mM pyruvate. In all cases, the cells were maintained at 37 °C in 5% CO_2_ atmosphere of 95% humidity. Cells were sub-cultured and the medium was changed once a week. In all cases, 0.25% trypsin, 0.25 mM ethylenediaminetetraacetic acid (EDTA) was used. Before and after the experiments, all cell lines were mycoplasma-free, as determined by the Hoechts DNA stain method [47].

### 3.3. Cytotoxicity Assay

A total of 5 × 10^3^ cells/well (200 µL of the culture medium described above) were seeded into a 96-well plate and incubated at 37 °C in a 5% CO_2_ and 95% humidity atmosphere for 24 h. A solution of each compound was prepared at a final concentration of 250 μM in DMSO (<1%). Successive 1:1 dilutions were performed, obtaining a total of sixteen solutions of concentrations ranging from 250 μM to 0.00762 μM, all of them in culture medium. Finally, a 200 µL aliquot of each of these last sixteen solutions was added to the wells. Cells were incubated at 37 °C for 48 h. The medium was then removed from the wells and 200 μL of MTT (3-(4,5-dimethylthiazol-2-yl)-2,5-diphenyltetrazolium bromide, 1 mg/mL final concentration) was added. After 4 h incubation in identical conditions, MTT was removed and 100 µL of dimethyl sulfoxide (DMSO) added. The absorbance at 560 nm was measured and recorded in a Fluostar Omega spectrophotometer.

Absorbances at each compound concentration were translated into inhibition percentages, I%, according to Equation (1):

(1) $$I\%\;=\;\left[1-\;\frac{A_{T}}{A_{C}}\right]\times 100$$

where *A_T_* and *A_C_* are the absorbance of treated and control cells, respectively.

IC_50_ values were obtained from a three-parameter fitting of the semi-logarithmic curves *(I%* as a function of the logarithm of the compound concentration) according to Equation (2):

(2) $$I\%\;=\frac{Imax}{\left[1\;+{\left(\frac{IC_{50}}{C}\right)}^{n}\right]}\;\times 100$$

where *Imax* is the maximum inhibition observed, IC_50_ is the compound concentration at which 50% of the cell population is death, *C* is the compound concentration at what the inhibition *I%* is observed and n is the slope of the curve at the IC_50_ value. The fitting was performed using GraphPad Prism v.8 software. All compounds were tested in three independent sets with triplicate points. The in vitro studies were performed in SACE (Support Service for Experimental Sciences, University of Murcia, Murcia, Spain) with biosecurity Level 2.

## 4. Conclusions

Of the six complexes analyzed in this work, two had been described previously and the other four were specifically designed and synthesized and fully characterized for this study. The cytotoxicity of all the new compounds was evaluated against three cell lines by means of an MTT assay. The results showed that Complexes I, IV and V (with ligands 4-cyanopyridine and pyridazine) had no cytotoxic effect against HeLa, MCF-7 or BGM cells, when they used IC_50_ concentrations lower than 250 μM. By contrast, Complexes II, III and VI (with the ligand L = aminophenol) were cytotoxic against at least two cell lines. However, while Complexes II and VI, (both with the ligand 2-aminophenol) were more cytotoxic against cancer cells, while Complex III (with the ligand 4-aminophenol) was more aggressive against healthy cells. All these findings justify further studies into the use of ruthenium compounds as promising anticancer agents due to their unique and versatile biochemical properties, that serve as alternatives to cisplatin and its derivatives.

## Figures and Tables

**Table 1:** Color, yield, analysis, exact mass and melting point (M.P.) for complexes III–VI. (a. calculated values in parenthesis; b. decomposition temperatures from the thermogravimetric curves.).

Complex	Color	Yield	Analytical Data ^a^			Mass Data	M.P. ^b^
		(%)	C	H	N	Fragments	
III	Yellow brown	95	45.16 (46.27)	4.87 (5.10)	3.33 (3.37)	281.0131 (281.1013) [M-*p*-cymene] 344.0595 (344.4141) [M-2Cl]	220
IV	Orange	97	42.61 (43.53)	4.35 (4.70)	7.17 (7.25)	351.0176 (350.8289) [M-Cl] 315.0444 (315.3762) [M-2Cl]	195
V	Light brown	74	40.57 (42.35)	3.46 (3.55)	8.55 (8.98)	489.0334 (489.7057) [M-*p*-cymene] 144.9649 (144.9642) [PF_6_]	157
VI	Dark brown	45	40.91 (41.68)	4.20 (4.45)	4.33 (4.42)	Decompose into solution	208

**Table 2:** Some relevant IR data (cm^−1^; Nujol mulls) for complexes III–VI.

Complex	v(N–H)	v(Ru–Cl)	Others
**III**	3210 m	278 s, 249 sh	3256 m ν(O–H)
**IV**		288 s, 278 s	
**V**		293 s,	2246 s ν(C≡N) 849 s,br ν(PF_6_^−^) 557 versus ν(PF_6_^−^)
**VI**	3148 w	269 w	846 s, br ν(PF_6_^−^) 559 versus ν(PF_6_^−^)

**Table 3:** NMR-1H data of the complexes III–VI. (J = Hz).

Complex	^1^H δ(SiMe4)	Ligand Structure
**III**	7.29 (ddt, 2H, H^3^ + H^4^) 6.80 (m, 2H, H^2^ + H^5^) 5.48 (s, 2H, NH_2_) 5.05–5.11(dd, 4H, C_6_H_4_-, J = 6.0) 2.70 (sept, 1H, -CH(CH_3_)_2_, J = 7.2) 2.05 (s, 6H, CH_3_-C_6_H_4_-) 1.17 (d, 6H, -CH(CH_3_)_2_, J = 6.8)	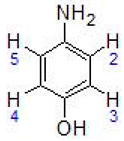
**IV**	9.20 (dt, 1H, H^6^, J = 8.0) 8.96 (m,1H, H^3^) 7.55 (m, 2H, H^4^ + H^5^) 5.37–5.62 (dd, 4H, C_6_H_4_-, J = 6.2) 3.09 (sept, 1H, -CH(CH_3_)_2_, J = 7.2) 2.28 (s, 3H, CH_3_-C_6_H_4_-) 1.24 (d, 6H, -CH(CH_3_)_2_, J = 6.8)	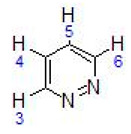
**V**	9.22 (dd, 2H, H^1^ + H^4^, J = 1.2) 7.73 (d, 2H, H^2^ + H^3^, J = 1.6) 5.66–5.95 (dd, 4H, C_6_H_4_-, J = 6.2) 2.57 (sept, 1H, -CH(CH_3_)_2_, J = 6.8) 1.76 (s, 3H, CH_3_-C_6_H_4_-) 1.50 (d, 6H, -CH(CH_3_)_2_, J= 6.8)	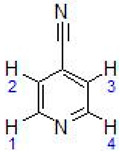
**VI**	Decompose into solution.	

**Table 4:** Cytotoxicity expressed as an IC_50_ mean values (μM) of the complexes synthesized and cisplatin (from the literature) exposed to HeLa, MCF-7 and BGM cells for 48 h.

Compound		IC_50_ (μM)	
	HeLa	MCF-7	BGM
**Complex I**	>250	>250	>250
**Complex II**	82.9 ± 0.67	28.7 ± 1.8	>250
**Complex III**	171.1 ± 2.1	75.8 ± 2.3	59.6 ± 1.9
**Complex IV**	>250	>250	>250
**Complex V**	>250	>250	>250
**Complex VI**	57.6 ± 4.0	24.9 ± 4.3	95.0 ± 1.7
**cisplatin**	67.6 ± 2.0 [45]	7.15 ± 0.1 [46]	5.45 ± 0.2 [46]

**Table 5:** Cytotoxicity expressed as IC_50_ mean values (µM) of the ligands exposed to HeLa, MCF-7 and BGM cells for 48 h.

Ligand		IC_50_ (μM)	
	HeLa	MCF-7	BGM
**4-cyanopyridine**	>250	>250	>250
**Pyridazine**	>250	>250	>250
**L = 2-aminophenol**	>250	131.33 ± 0.15	>250
**L = 4-aminophenol**	>250	>250	>250

## Supplementary Materials

The supplementary materials are available online. Figure S1: 1H NMR spectrum of complex III, Figure S2: 1H NMR spectrum of complex IV, Figure S3: 1H NMR spectrum of complex V, Figure S4: The 1H-1H COSY of complex IV, Figure S5: Mass spectrum of complex III, Figure S6: Mass spectrum of complex IV, Figure S7: Mass spectrum of complex V.
