# Supplementary material for: p-Cymene Complexes of Ruthenium(II) as Antitumor Agents
Source: Molecules. 2020 Oct 31;25(21):5063. doi: 10.3390/molecules25215063 (PMC7662397; doi:10.3390/molecules25215063)
Supplement: Supplementary file 1 [file molecules-25-05063-s001.pdf]

Supporting Information for

p-cymene complexes of ruthenium(II) as antitumor agents

María Angeles Pujante-Galián [1], Sergio A. Pérez [2], Mercedes G. Montalbán [2,*], Guzmán Carissimi [2], Marta G. Fuster [2], Gloria Víllora and Gabriel García [1]

[1] Inorganic Chemistry Department, Faculty of Chemistry, Regional Campus of International Excellence "Campus Mare Nostrum", University of Murcia, 30071, Murcia, Spain; mariaangeles.pujante@um.es (M. A. P.-G.), ggarcia@um.es (G. G.).

[2] Chemical Engineering Department, Faculty of Chemistry, Regional Campus of International Excellence "Campus Mare Nostrum", University of Murcia, 30071, Murcia, Spain; sa.perezhenarejos@um.es (S. A. P.), guzmanaugusto.carissimin@um.es (G. C.), marta.g.f@um.es (M. G. F.), gvillora@um.es (G. V.)

* Correspondence: mercedes.garcia@um.es; Tel.: +34-868887926 (M. G. M.)

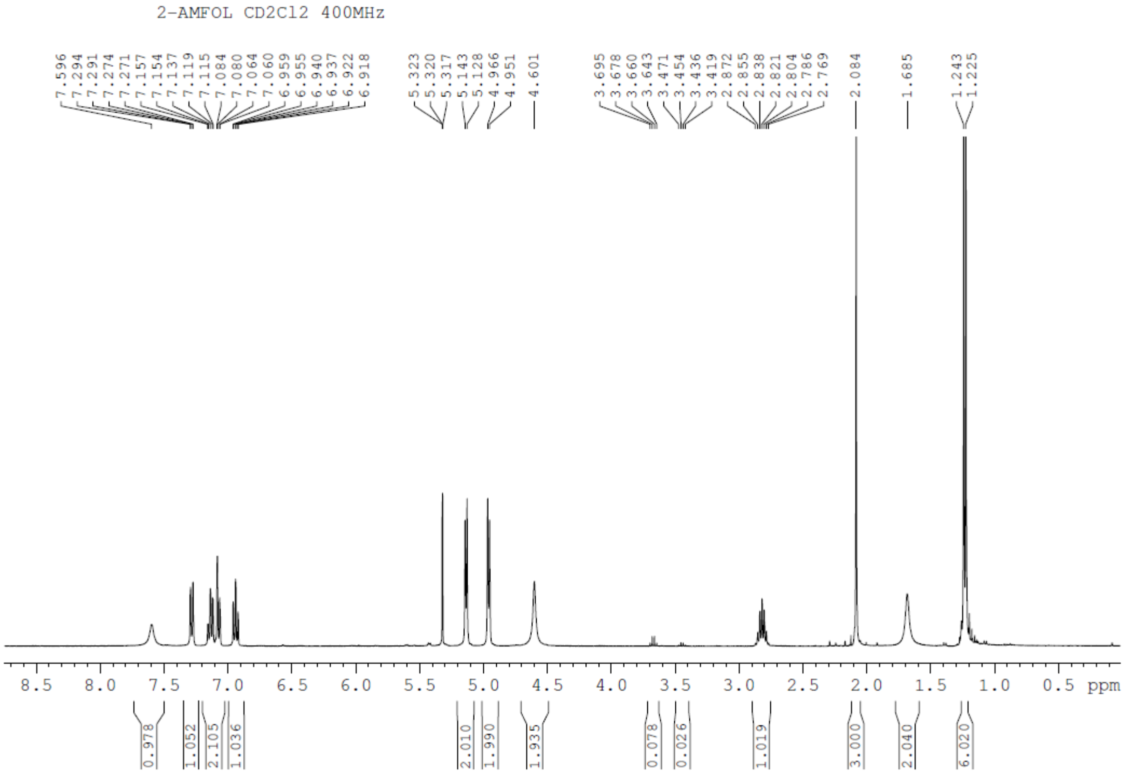

Figure S1. 1H NMR spectrum of complex III.

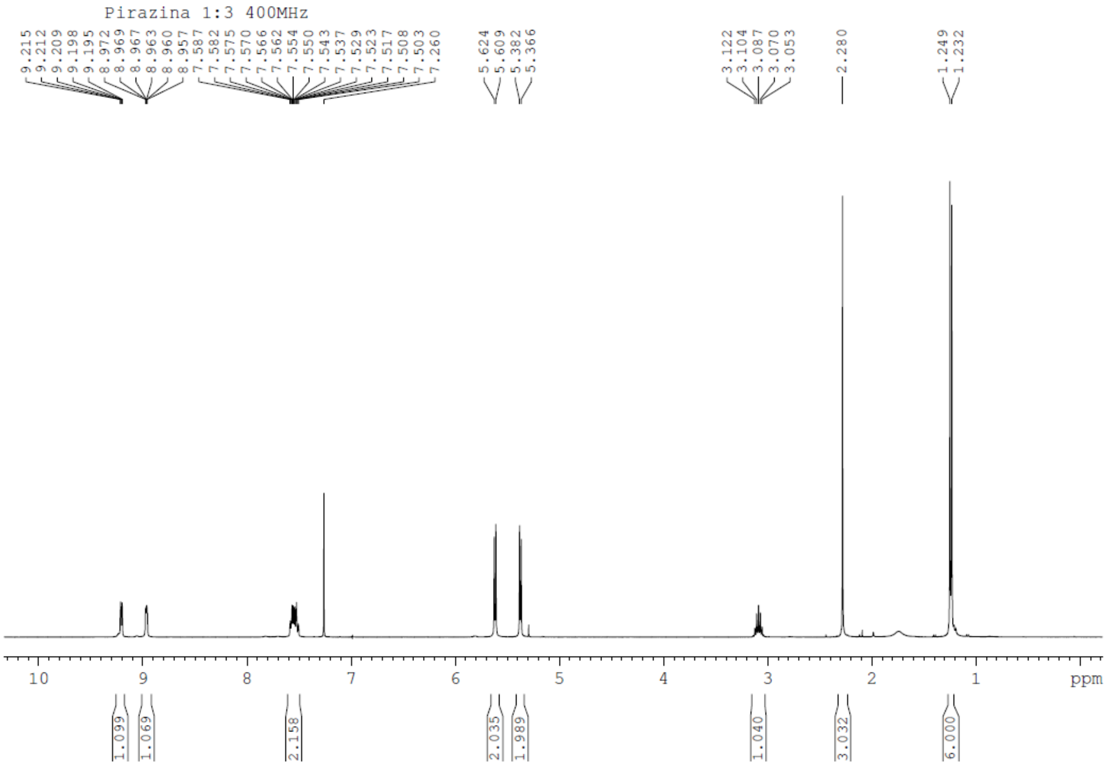

Figure S2. 1H NMR spectrum of complex IV.

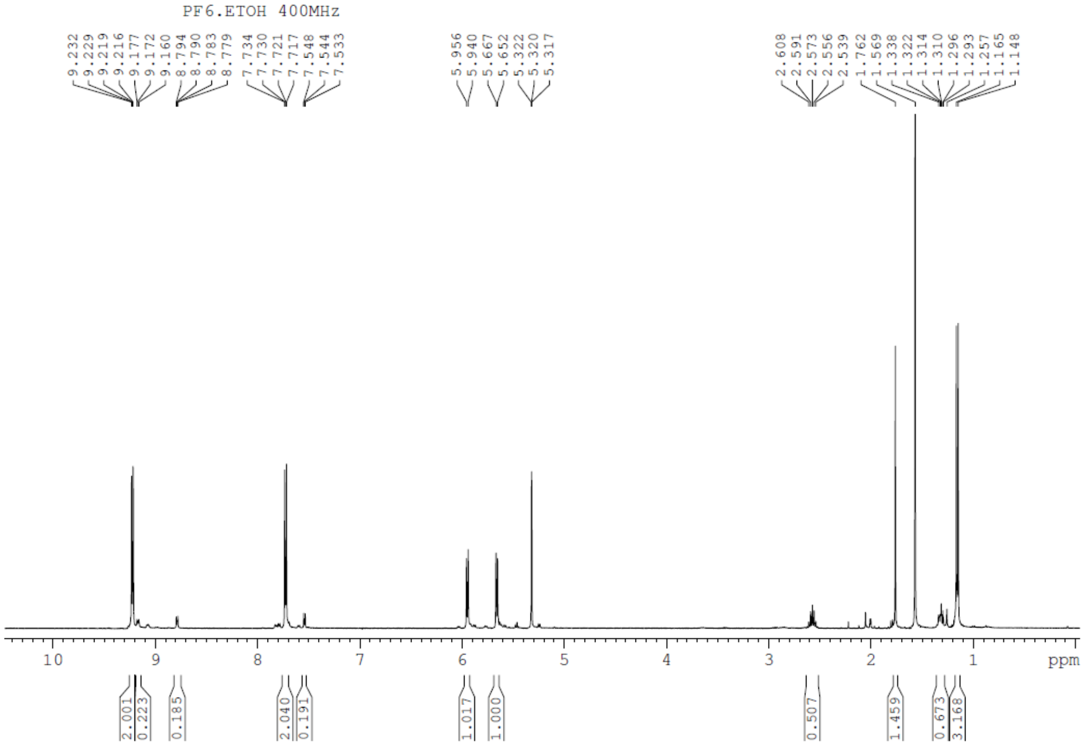

Figure S3. 1H NMR spectrum of complex V.

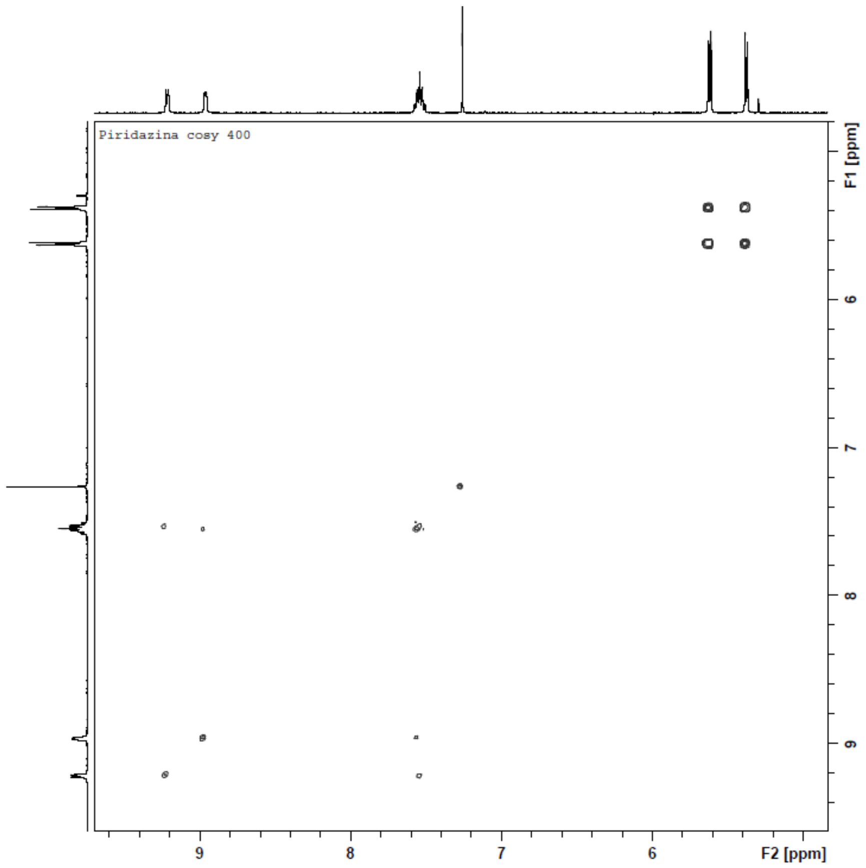

Figure S4. The 1H-1H COSY of complex IV.

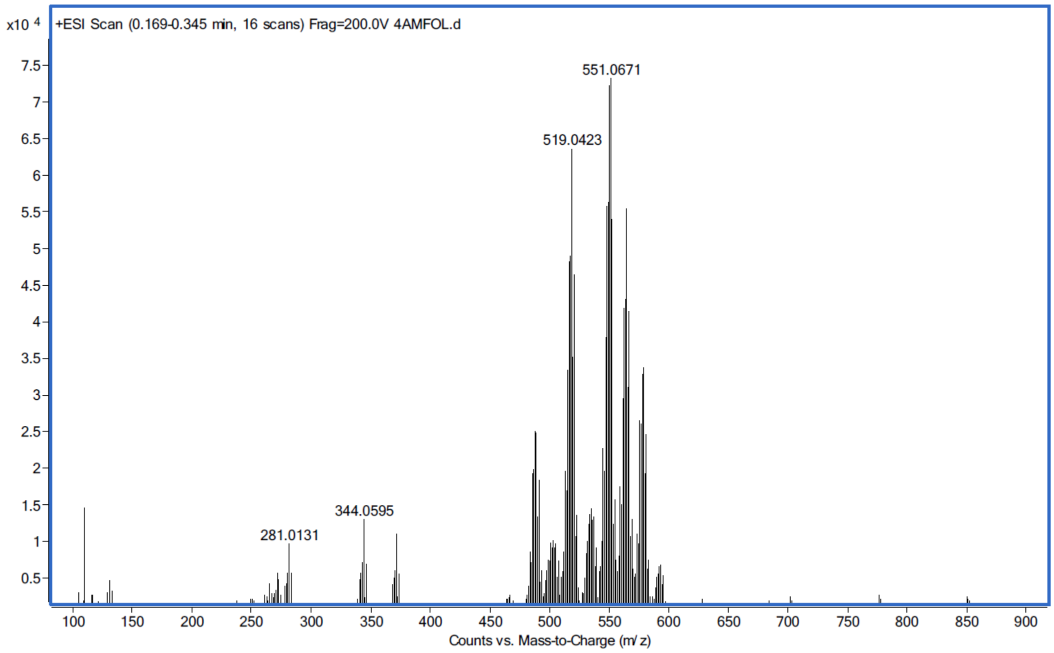

Figure S5. Mass spectrum of complex III.

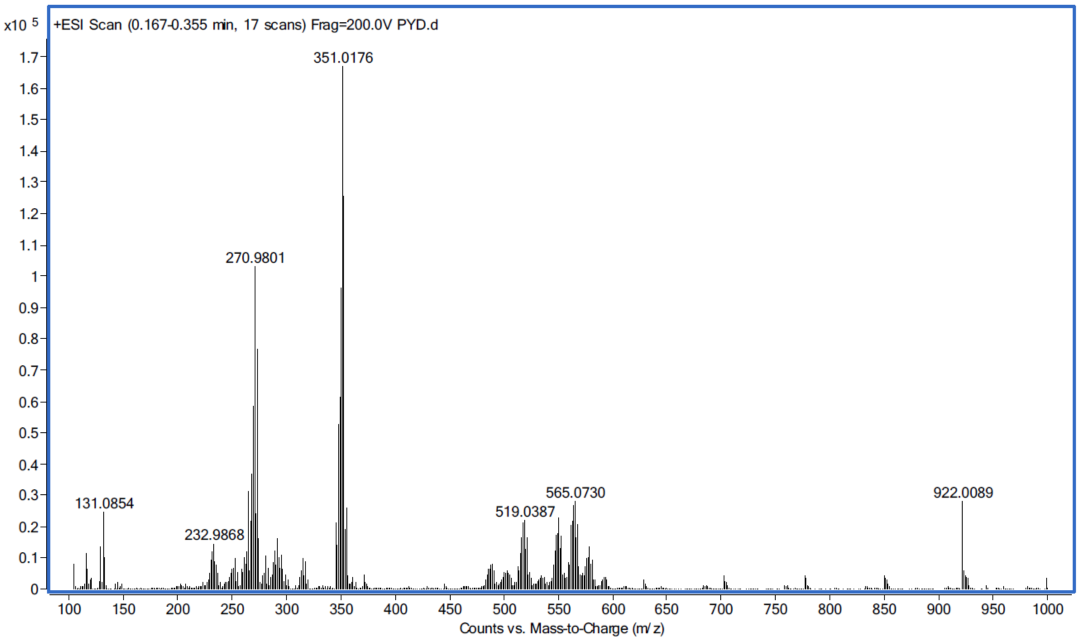

Figure S6. Mass spectrum of complex IV.

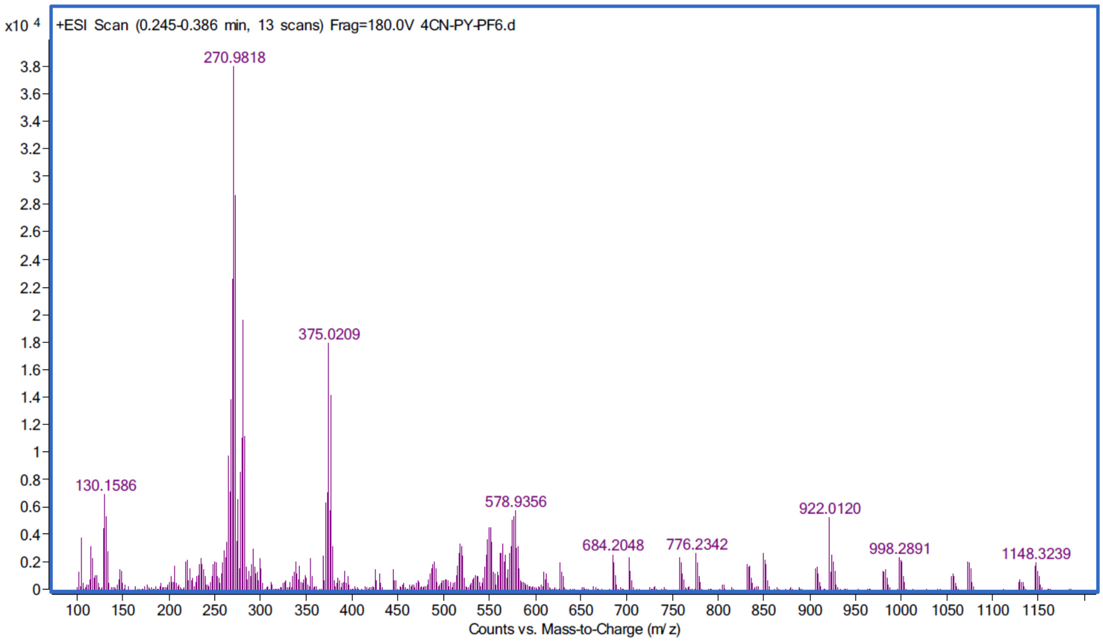

Figure S7. Mass spectrum of complex V.
